# Appearance of cysts and capillary non perfusion areas in diabetic macular edema using two different OCTA devices

**DOI:** 10.1038/s41598-020-57680-w

**Published:** 2020-01-21

**Authors:** Mariacristina Parravano, Eliana Costanzo, Enrico Borrelli, Riccardo Sacconi, Gianni Virgili, SriniVas R. Sadda, Fabio Scarinci, Monica Varano, Francesco Bandello, Giuseppe Querques

**Affiliations:** 1 IRCCS - Fondazione Bietti, Rome, Italy; 2 Department of Ophthalmology, IRCCS Ospedale San Raffaele, University Vita-Salute, Milan, Italy; 3 Department of Neurosciences, Psychology, Drug Research and Child Health (NEUROFARBA), University of Firenze and AOU Careggi, Firenze, Italy; 4 Doheny Image Reading Center, Doheny Eye Institute, Los Angeles, California USA; 5 Department of Ophthalmology, David Geffen School of Medicine at UCLA, Los Angeles, California USA

**Keywords:** Medical research, Engineering

## Abstract

The aim of this paper was to distinguish the appearance of cysts and non-perfusion areas (NPAs) in diabetic macular edema (DME) using two different Optical Coherence Tomography Angiography (OCTA) devices. In this study, patients underwent OCTA using the AngioVue XR Avanti Spectral Domain (SD) OCTA and the PLEX Elite 9000 Swept-Source (SS) OCTA. Foveal and extrafoveal regions of interest (ROI), defined as any area with an altered flow signal comparing to the surrounding retina, were selected in superficial and deep capillary plexus (SCP and DCP). ROI reflectivity were classified as hypo-reflective or hyper-reflective. Foveal ROI were analyzed to detect suspended scattering particles in motion (SSPiM). Thirty-seven DME eyes were included. A larger number of ROIs were found in SCP (55 vs 39) and DCP (60 vs 49) using PLEX Elite 9000 vs AngioVue. The majority of ROIs were hypo-reflective with both instruments, while slightly more hyper-reflective ROIs (grey) were detected with the PLEX Elite, more likely to be cysts. The hyporeflective ROIs could be NPAs or cysts with both devices. Moreover, PLEX Elite 9000 identified SSPiM in more foveal ROIs than the AngioVue in the SCP (p = 0.005) and in the DCP (p = 0.027). In conclusion, NPAs and cysts may show variable appearances using different OCTA devices. Hyperreflective ROIs generally correspond to cysts, hyporeflective ROIs can be either cysts or NPAs. The SS-OCTA seems to detect SSPiM more frequently than the SD-OCTA.

## Introduction

Diabetic macular edema (DME) is one of the most important causes of visual impairment in the working-age population in industrialized countries^1^. DME is directly associated with a breakdown of the blood retinal barrier (BRB)^2^, that determines fluid accumulation and an increase in retinal thickness^3^. The clinical and anatomical features of DME are the presence of hard exudates, microaneurysms, hyperreflective intraretinal deposits and cysts that are mostly confined to the outer nuclear and Henle layers^3^. Traditionally, these alterations have been invasively described using fluorescein angiography. In recent years, the evaluation of these retinal characteristics has been revolutionized by the introduction of optical coherence tomography angiography (OCTA). The combination of these techniques, integrated with the structural information provided by structural OCT, has improved the identification and follow-up of microaneurysms, non-perfusion areas (NPAs), and intra and sub-retinal fluid^4–6^.

Noteworthy, De Carlo *et al*.^7^ differentiated the appearance of cysts and NPAs based on enface OCTA images. To do so, they used a spectral domain OCTA (SD-OCTA) device (AngioVue RTVue XR Avanti, Optovue, Fremont, CA, USA), that uses a processing algorithm termed split-spectrum amplitude-decorrelation algorithm (SSADA). In particular, the authors evaluated the OCTA signal voids at the level of superficial capillary plexus (SCP) and deep capillary plexus (DCP), concluding that the cystoid DME spaces were seen as oblong areas, devoid of signal, appearing black on enface OCTA angiograms. In contrast, NPAs were displayed as regions with a grey hue, bordered by adjacent capillaries, probably because of signal noise.

Since then, numerous OCTA devices have been developed. These devices have different wavelengths and acquisition strategies that can be broadly divided into SD-OCTA and swept source OCTA (SS-OCTA).

Recently, several studies^8–11^ have demonstrated that the blood flow parameters extracted from different devices are not interchangeable. Different OCTA instruments display different tissue reflectivity that influence the flow detection^8,10^.

Another important critical point was highlighted in 2017 by Kashani *et al*.^12^, that recognized a new OCTA feature in retinal vascular diseases, including cystoid diabetic macular edema, called suspended scattering particles in motion (SSPiM). SSPiM represents an extravascular OCTA signal related to varying degrees of hyperreflective material on structural OCT, likely due to Brownian motion of particles within the intraretinal fluid, that appears as flow on OCTA B-scans. Using an *in vitro* phantom, it has been demonstrated that there is a difference in OCTA signal detected inside the cysts between SSADA (algorithm of SD-OCTA AngioVue) and optical microangiography (OMAG) (algorithm used in SD-OCTA AngioPlex and SS-OCTA PLEX Elite 9000, Carl Zeiss Meditec Inc., Dublin, CA, USA) model^12^.

The aim of our study was to compare the appearance of cysts and NPAs in eyes with diabetic macular edema (DME) using two different OCTA devices.

## Results

Thirty-seven DME eyes of 20 type 2 diabetic patients (7 females, 13 males) were enrolled. Three eyes from 3 patients were excluded from the analysis because of the absence of macular edema. Of the enrolled eyes, 16 were treatment-naïve, while 21 eyes were previously treated with anti-VEGF or dexamethasone intravitreal injections. Mean ± SD age was 69.2 ± 8.4 years.

Mean ± SD BCVA was 59.5 ± 15.2 ETDRS letters (20/63 Snellen equivalent, with a range from 20/20 to 20/400). Mean ± SD CRT as determined on the macular map was 520.1 ± 118.5 µm.

### Region of interest identification with OCTA

The unmasked observer identified a larger number of candidate ROIs using the PLEX Elite 9000 vs the AngioVue for both the SCP (55 vs. 39) and DCP (60 vs. 49), meaning that 41% and 22% more ROIs were identified with PLEX Elite 9000.

There was a good to almost perfect agreement between the two masked observers in assessing ROIs reflectivity on OCTA images. Specifically, for ROIs in the SCP layer raw agreement was 0.92 and 0.98 and Cohen’s kappa was 0.83 and 0.94, for AngioVue and PLEX Elite 9000, respectively. For ROIs in the DCP layer, raw agreement was 0.98 for both AngioVue and PLEX Elite 9000, while Cohen’s kappa was 0.85 and 0.94 for the two devices, respectively. Therefore, we used the rating of the senior unmasked observer to compare the OCT devices with regards to the ROIs assessment.

### Characteristics of cysts and NPAs with OCTA

**Figure 1:**
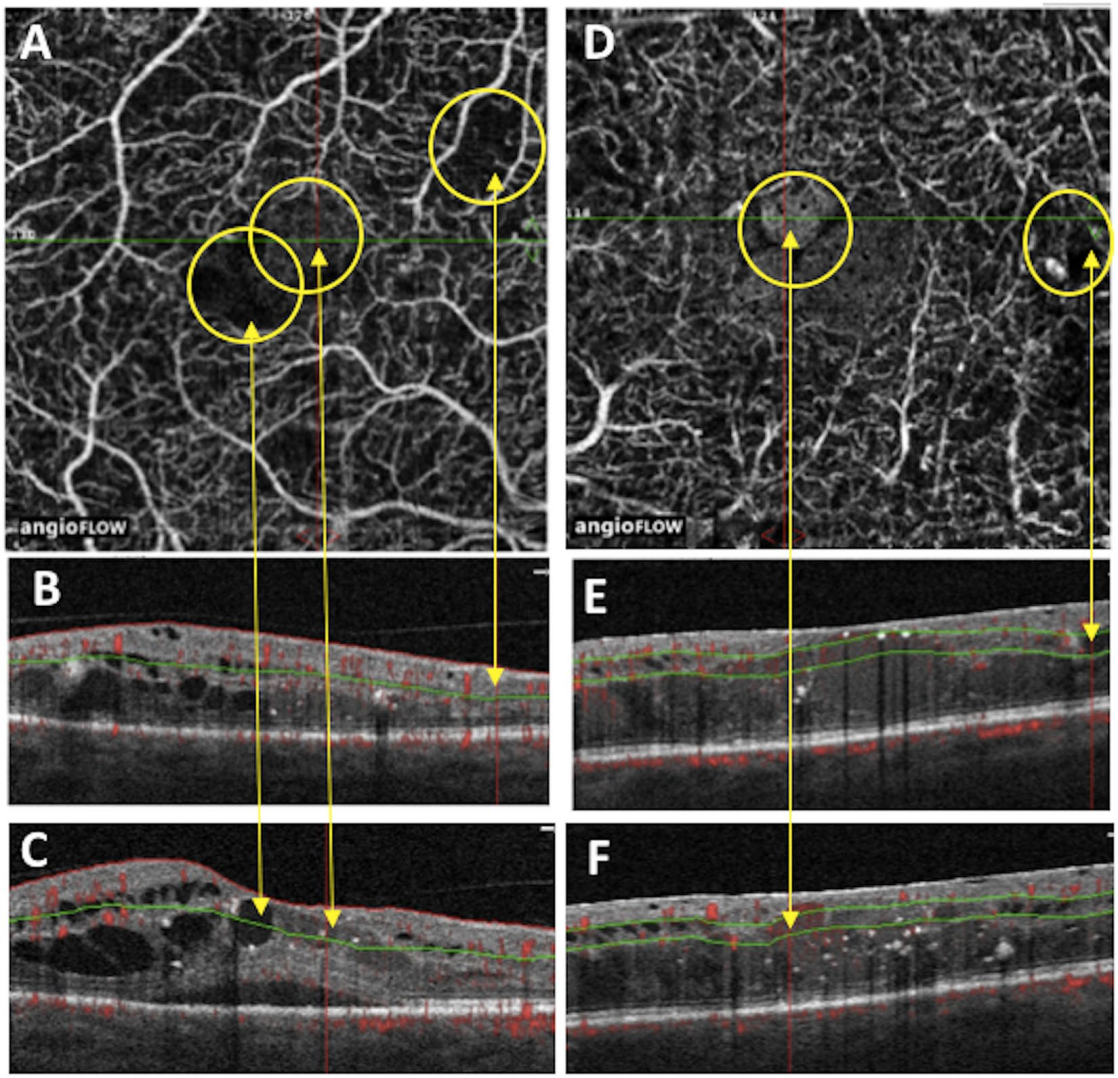
ROIs in SCP and DCP imaged by AngioVue. In (**A**) SCP scans with visualization of 3 ROIs (yellow circles): the rightmost hyporeflective ROI (rightmost yellow circle) that corresponded to NPA on structural B-scan OCT (visualized in (**B**) pointed out by yellow arrow), the middle hyperreflective ROI (middle yellow circle) that corresponded to cyst on structural-scan OCT (visualized in (**C**) pointed out by yellow arrow), the leftmost hyporeflective ROI (leftmost yellow circle) that corresponded to cyst on structural B-scan OCT (visualized in (**C**) pointed out by yellow arrow). In (**D**) DCP of 2 ROIs (yellow circles): the rightmost hyporeflective ROI (rightmost yellow circle) that corresponded to cyst on structural B-scan OCT (visualized in (**E**) pointed out by yellow arrow), the leftmost hyperreflective ROI (leftmost yellow circle) that corresponded to cyst on structural-scan OCT (visualized in F, pointed out by yellow arrow).

**Figure 2:**
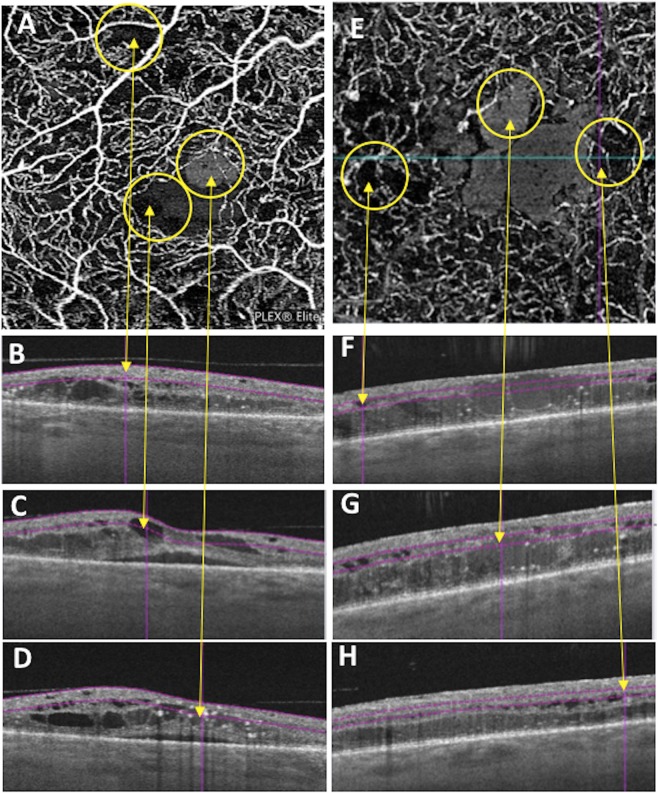
ROIs in SCP and DCP imaged by PlexElite. In (**A**) SCP scans with visualization of 3 ROIs (yellow circles): the leftmost hyporeflective ROI (leftmost yellow circle) that corresponded to NPA on structural B-scan OCT (visualized in (**B**) pointed out by yellow arrow), the middle hyporeflective ROI (middle yellow circle) that corresponded to cyst on structural-scan OCT (visualized in (**C**) pointed out by yellow arrow), the rightmost hyperreflective ROI (rightmost yellow circle) that corresponded to cyst on structural B-scan OCT (visualized in (**D**) pointed out by yellow arrow). In (**E**) DCP of 3 ROIs (yellow circles): the leftmost hyporeflective ROI (leftmost yellow circle) that corresponded to cyst on structural B-scan OCT (visualized in (**F**) pointed out by yellow arrow), the middle hyperreflective ROI (middle yellow circle) that corresponded to cyst on structural-scan OCT (visualized in (**G**) pointed out by yellow arrow), the rightmost hyporeflective ROI (rightmost yellow circle) that corresponded to NPAs on structural-scan OCT (visualized in (**H**) pointed out by yellow arrow).

The large majority of ROIs were hyporeflective (i.e. black) with both instruments while slightly more hyperreflective (i.e. grey) ROIs were detected with the PLEX Elite 9000 vs AngioVue both at the SCP [18% (10/55) vs. 8% (3/39)] and the DCP [17% (11/60) vs. 8% (4/49)]; however, such differences did not reach nominal statistical significance (Figs. 1 and 2).

Table 1 presents the cross-tabulation of OCTA and structural OCT findings regarding ROI classification in the SCP and DCP layers.

**Table 1:** Cross-classification of Regions Of Interest (ROIs) identified as cysts or Non-Perfused Areas (NPAs) with structural OCT as compared with grey or black lesions with OCT Angiography using all ROIs identified using PLEX ELITE 9000 and ANGIOVUE.

	SCP			total	*% cyst*
		cyst	NPA		
PLEX ELITE 9000	grey	7	3	10	*70%*
	black	16	29	45	*36%*
ANGIOVUE	grey	2	1	3	67%
	black	20	16	36	56%
	**DCP**				
		**cyst**	**NPA**	**total**	**%** ***cyst***
PLEX ELITE 9000	grey	11	0	11	100%
	black	34	15	49	69%
ANGIOVUE	grey	4	0	4	100%
	black	41	4	45	91%

Hyporeflective ROIs could be either NPAs or cysts with both OCTA devices. In particular PLEX Elite 9000 identified a larger number of ROIs than AngioVue and a higher number of hyporeflective ROIs compared with AngioVue. Regarding ROIs in the SCP layer, 16/45 (36%) hyporeflective ROIs were cysts with PLEX Elite 9000 as compared to 20/36 (56%) with the AngioVue. Hyperreflective ROIs were found to be cysts in 7/10 (70%) vs. 2/3 (67%) respectively. The differences between devices were not statistically significant, but comparisons are made difficult by the small number of hyperreflective cysts, particularly with the AngioVue.

In the DCP, hyporeflective ROIs were mostly cysts with both PLEX Elite 9000 and AngioVue: 34/49 (69%) and 41/45 (91%), respectively (p = 0.001). This means that PLEX Elite 9000 identified 15 hyporeflective ROIs corresponding to NPA as compared to only 4 with AngioVue. On the other hand, hyperreflective ROIs were cysts in all cases with both devices, and, similarly, PLEX Elite 9000 identified more hyperreflective ROIs than the AngioVue (11 vs. 4) as reported above.

### Characteristics of SSPiM with OCTA and structural OCT

**Figure 3:**
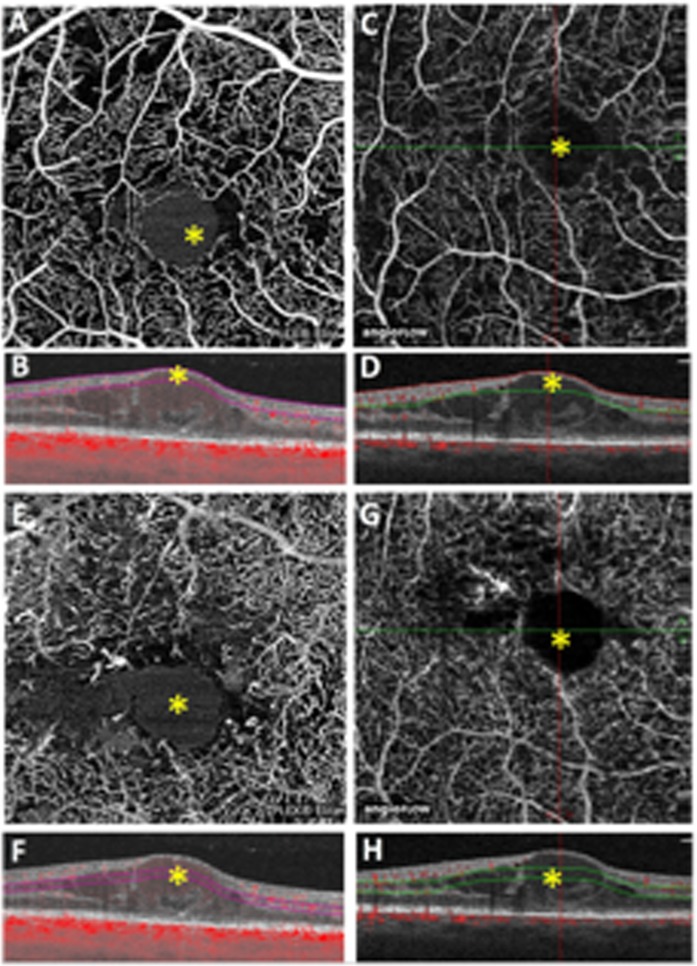
Foveal ROIs in SCP and DCP acquired with PlexElite and AngioVue. In (**A**) hyperreflective central ROI (yellow*) imaged by PlexElite at the level of SCP with corresponding structural B-scan OCT with flow (**B**) and visualization of SSPiM. In (**C**) the same ROI visualized in A that appeared as hyporeflective imaged by AngioVue with its corresponding structural B-scan OCT with flow (**D**) without visualization of SSPiM. In (**E**) hyperreflective central ROI (yellow*) imaged by PlexElite at the level of DCP with corresponding structural B-scan OCT with flow (**F**) and visualization of SSPiM. In (**G**) the same ROI visualized in (**E**) that appeared as hyporeflective imaged by AngioVue with its corresponding structural B-scan OCT with flow (**H**) without visualization of SSPiM.

Because the interpretation of these data is made difficult by the lower number of ROIs that could be identified with AngioVue, regarding our objective of investigating the structural association of SSPiM, we restricted the analysis to a cross-tabulation of 37 paired foveal ROIs that could be identified with both devices, as shown in Fig. 3.

PLEX Elite 9000 identified SSPiM in more ROIs than AngioVue in both the SCP (16/37, 43% vs. 9/37, 24%, p = 0.005) and the DCP (17/37, 46% vs. 8/37, 22%; p = 0.027) (Table 2). Moreover, SSPiM corresponded to cysts in about 80% of ROIs for the PLEX Elite 9000 and in about 60% of ROIs for the AngioVue in both the SCP and DCP, (p = 0.170 and p = 0.278, respectively). In discordant cases analysis (Fig. 3), the PLEX Elite 9000 showed a higher SSPiM detection rate both in the SCP and the DCP in comparison with the AngioVue. In particular all discordant foveal ROIs which were hyperreflective in the PLEX Elite 9000 and hyporeflective with AngioVue (8 SCP and 10 DCP) showed SSPiM with the PLEX Elite 9000, as compared with only 1 and 2 ROIs for the AngioVue, respectively. On the other hand, in fewer discordant ROIs where PLEX Elite 9000 was hyporeflective and AngioVue was hyperreflective (5 SCP and 1 DCP) only one case in each layer showed SSPiM with both devices.

**Table 2:** Cross-classification of Regions Of Interest (ROIs) identified as cysts or Non-Perfused Areas (NPAs) with structural B scan with flow as compared with the presence or absence of Suspended Scattering Particles in Motion (SSPiM) with OCT Angiography using paired ROIs identified using both PLEX ELITE 9000 and ANGIOVUE.

	SCP			total	*% cyst*
		cyst	NPA		
PLEX ELITE 9000	SSPiM	13	3	16	*81%*
	no	0	21	21	*0%*
ANGIOVUE	SSPiM	5	4	9	56%
	no	5	23	28	18%
	**DCP**				
		**cyst**	**NPA**	**total**	***% cyst***
PLEX ELITE 9000	SSPiM	14	3	17	82%
	no	0	20	20	0%
ANGIOVUE	SSPiM	5	3	8	63%
	no	0	29	29	0%

## Discussion

The structural OCT characteristics of DME have been widely studied; however, the features of DME on OCTA images and the characteristics of the internal reflectivity of cysts are not yet fully understood and there is no consensus on their interpretation^13–15^. Our study demonstrates that OCTA devices are not interchangeable for the classification of the reflectivity of the signals of cysts and NPAs in eyes with DME. Moreover, the devices showed a different ability in detecting SSPiM in ROIs, which was found to be a marker for cysts, particularly with the PLEX Elite 9000.

A recent study from Farci *et al*.^16^, correlated the OCTA signal with the enface OCT signal inside cystoid spaces in diabetic macular edema and retinal vein occlusion, using SSADA devices. The authors found a low intensity OCTA signal in the cystoid spaces, reopening questions regarding the nature of this signal, if it is due to a corpusculated material or to an artifact detected by the instruments.

In 2016, a pioneering study from De Carlo *et al*.^7^, described the cystoid spaces of DME patients as the black areas on OCTA, in contrast with the grey areas representing NPAs.

Our study provides a novel approach to the characterization of DME using OCTA, through the comparison between two different devices with different acquisition algorithms, correlating the enface OCTA with each other and with corresponding B-Scan OCT with flow, not only for the definition of cysts and NPAs but also for the detection of SSPiM.

We observe that the same DME characteristics are depicted differently by different devices which use different OCTA algorithms (SSADA vs OMAG). The first difference was that PLEX Elite 9000 recognized more numerous candidate ROIs than the AngioVue. There may be several explanations for this. An important observation during analysis was the frequent errors in automatic segmentation provided by both devices, due to vessel displacement caused by the presence of cysts. The manual segmentation procedures differed between devices, and the PLEX Elite 9000 segmentation’s lines proved to be more easy to modify and reposition in the correct location compared to the AngioVue for both the SCP and DCP.

In our study, the ROIs were classified as hyporeflective (i.e. black) or hyperreflective (i.e. grey). The large majority of extrafoveal ROIs were hyporeflective with both instruments and could correspond to both cysts and NPAs in both plexa. Using PLEX Elite 9000, a greater number of extrafoveal hyperreflective ROIs than AngioVue were detected, at the level of SCP and DCP, corresponding to cysts.

A previous aforementioned study^7^, described the aspect of cysts and NPAs in DME, using SSADA OCTA, analyzing the SCP and DCP. The authors noted that while cystoid spaces appeared as “black” regions, devoid of signal, with an oblong shape, NPAs appeared as grey regions with some internal signal noise and bordered by capillaries. In line with this study we found that, using the AngioVue (SSADA), 56% of hyporeflective ROIs in the SCP and 91% in DCP corresponded to cysts. However, the results were quite different with the PLEX Elite 9000 (OMAG) which detected a lower number of hyporeflective ROIs that corresponded to cysts.

The most important difference compared to prior studies was apparent in the analysis of hyperreflective ROIs. Using both devices, grey ROIs were more likely to be cysts, and not NPAs as described by De Carlo^7^. About 70% of hyperreflective ROIs in the SCP and 100% in the DCP corresponded to cysts, as shown in Table 1. This difference could be explained by technical characteristics of the device used for the analysis and by the position of the borders of the enface slabs. As demonstrated by Kashani *et al*.^12^, the signal detected in SSADA and OMAG models, were different and the OMAG showed more internal reflectivity than SSADA models.

In our study, this factor, which could impact the characteristics of fluid inside cysts, was more evident in the analysis of foveal cysts. Comparisons using the foveal region are more reliable as they are less affected by segmentations’ errors in both the SCP and DCP. In line with Kashani *et al*.^12^, we found that the presence of hyperreflective foveal cysts is related to SSPiM, more frequently using PLEX Elite 9000.

Limitations of our study include the small sample size and the inclusion of both, treated and treatment-naïve DME patients. Another limitation is the difference in ROIs detected with both instruments, with more ROIs identified with the PLEX Elite 9000. However, this was due to difficulties with optimizing segmentation with the AngioVue device, and only the ROIs that were well segmented were included in the final analysis.

In conclusion, we found that OCTA devices differ regarding the identification of ROIs which may correspond to cysts or NPAs. A larger number of potential ROIs was identified with the PLEX Elite 9000 which could also detect SSPiM more efficiently than the Optovue Avanti. Our study suggests that hyperreflective ROIs generally correspond to cysts, but hyporeflective ROIs are more numerous and may correspond to cysts or NPA. These observations could be of value in the training and development of future artificial intelligence models to classify OCT and OCTA images in eyes with DME.

## Methods

In this cross-sectional observational study, patients with type 2 diabetes and DME complicating mild, moderate or severe non-proliferative diabetic retinopathy (DR), according to the modified Early Treatment Diabetic Retinopathy Study (ETDRS) retinopathy severity scale^17^, were retrospectively collected and analyzed at the Department of Ophthalmology, IRCCS-Fondazione Bietti, Rome, between March 15, 2018 and October 15, 2018.

This study was approved by the Institutional Review Board of the IRCCS-Fondazione Bietti, and followed the tenets of the Declaration of Helsinki. Written informed consent was obtained from all participants.

Exclusion criteria were: presence of macula edema secondary to other causes than DR (e.g. retinal vascular occlusion), diagnosis of other macular diseases, including central serous chorioretinopathy, vitreoretinal interface diseases or age-related macular degeneration. Patients with significant cataract, graded above NO3 or NC3^18^, were excluded. Poor quality images with a signal strength index (SSI) lower than 6 for the PLEX Elite and with a SSI lower than 50 for AngioVue or with significant motion artifacts (seen as large dark lines on the enface angiograms) were also excluded.

All patients received a complete ophthalmologic examination, which included the measurement of best corrected visual acuity (BCVA) using ETDRS charts, intraocular pressure (IOP), and dilated fundus examination.

### Imaging

All patients underwent structural SD-OCT using the Spectralis (Heidelberg Engineering, Heidelberg, Germany) and OCTA imaging using both SD-OCTA AngioVue XR Avanti and SS-OCTA PLEX Elite 9000 devices.

The mean central retinal thickness (CRT) was automatically measured in the macular map centered on the fovea, using instrument software (Heidelberg Spectralis version 1.10.2.0, Heidelberg Engineering, Heidelberg, Germany). All B-scan images were checked for errors in automatic segmentation and manual correction was made for all the identified segmentation errors.

Images were acquired with the XR Avanti OCTA instrument (Optovue Inc., Fremont, California, USA)^19^ with a 3 × 3-mm scanning area, centered on the fovea. Using the instrument’s automatic segmentation algorithm, the enface OCTA images were segmented to define the SCP and DCP. SCP upper limit was at the level of the inner limiting membrane (ILM) and lower limit was 9 micron below the outer border of inner plexiform layer (IPL); DCP was defined as a slab spanning between 9 micron below the outer border of IPL and 9 micron above the outer border of outer plexiform layer (OPL).

Patients underwent SS-OCTA imaging using the PLEX Elite 9000 device^8^ which uses a swept laser source with a central wavelength of 1050 nm and a bandwidth of 100 nm. This instrument has an axial resolution of approximately 5 microns and a lateral resolution estimated at approximately 14 μm. For each eye in the study, OCTA images using the 3 × 3 mm scan pattern were acquired, and using the segmentation algorithm by the built-in software the enface OCTA images were segmented to define SCP and DCP. The automatic SCP slab was segmented between ILM and IPL; for DCP the upper limit was at the level of IPL and the lower limit was defined by the OPL.

To avoid automatic errors due to vessel displacement caused by cysts, thinner slabs were also obtained with customized settings and were moved progressively from the outer retina to the inner plexiform layer with manual segmentation used to better visualize the plexa. The manual segmentation capabilities differed between the devices; with the AngioVue OCTA software version used in our study, it is possible the slab boundaries inward or outward, but is not possible to change the profile of the boundary lines; in contrast with the PLEX Elite 9000, it is possible to customize the width of the slab and also adjust the segmentation boundary position at any A-scan location.

After manual segmentation of the slabs, region of interests (ROIs) on enface OCTA images of the SCP and DCP acquired with the two devices were selected by one unmasked expert observer who verified the presence of cysts or NPAs on the corresponding structural OCT (Fig. 1e2), no other features as exudates, microaneurysms or hemorrhages were included in the analysis.

ROIs were selected in each 3 × 3 enface OCTA angiogram acquired with both devices. ROIs were defined as any area with an altered flow signal (hypo or hyper-reflective) comparing to the surrounding retina. Only the regions which were correctly segmented were included in the masked analysis. Afterward, we studied SCP and DCP foveal ROIs, analyzing the altered flow signal inside the central cystoid space. These ROIs were less affected by segmentations’ errors. For each patient, two graders (EB and RS) analyzed the spatial correspondence of the ROIs between 3 × 3 enface OCTA images acquired with PLEX Elite 9000 and AngioVue in order to identify the corresponding ROIs that were used for paired subanalysis.

For foveal ROIs, the masked observers also graded the presence of SSPiM on structural B scan with flow overlay associated with the presence of an altered signal (hypo or hyper-reflective) on enface OCTA angiograms.

In case of disagreement, there was open adjudication between graders to generate a single consensus result for all cases.

### Statistics

Descriptive statistics were based on cross-tabulation of imaging features. A chi-square test was used to compare proportions. Interobserver agreement regarding the coding of grey vs. black ROIs was assessed using Cohen’s kappa. Analyses were conducted using Stata 15.1 (StataCorp, College Station, TX).

## Data Availability

The data used to support the findings of this study are available from the corresponding author upon request.
